# Developmental Heterochronies and Dioxygen Constraints in Teleostean Fishes

**DOI:** 10.3390/jdb14040044

**Published:** 2026-10-04

**Authors:** Guillaume Lecointre, Victor Robitaille, Anatole Tailleur, Lucas Royer, Valentin Rineau, Boris Rota, Mehdi Thabet, Dominique Chardard, Fabrice Teletchea

**Affiliations:** 1 Équipe “Homologies”, Institut de Systématique, Évolution, Biodiversité (ISYEB), UMR 7205 Muséum National d’Histoire Naturelle, Centre National de la Recherche Scientifique, École Pratique des Hautes Études, Université des Antilles, Sorbonne Université, CP50, Muséum National d’Histoire Naturelle, 57 rue Cuvier, 75005 Paris, France; victor.robitaille@icloud.com (V.R.); anatoletailleur@gmail.com (A.T.); 2 Équipe Diversification en Aquaculture Continentale, Laboratoire Animal et Agroécosystèmes (L2A), Faculté des Sciences et Technologies, Université de Lorraine, Entrée 1B, 4ème étage, BP 70239, 54506 Vandoeuvre-lès-Nancy, France; l.royer55@laposte.net (L.R.); dominique.chardard@univ-lorraine.fr (D.C.); 3 Centre de Recherche en Paléontologie—Paris (CR2P), UMR 7207 Centre National de la Recherche Scientifique, Muséum National d’Histoire Naturelle, Sorbonne Université, 4, Place Jussieu, Tour 46-56 5e étage, 75252 Paris, France; valentin.rineau@sorbonne-universite.fr; 4 Recifathome, 6b rue des Prés-Chalots, 25220 Roche-lez-Beaupré, France; contact@recifathome.com (B.R.); medy.thabet@gmail.com (M.T.); 5 Laboratoire Interdisciplinaire des Environnements Continentaux (LIEC), UMR 7360 Université de Lorraine, Centre National de la Recherche Scientifique, Université de Lorraine, 15 Avenue du Charmois, 54500 Vandœuvre-lès-Nancy, France; fabrice.teletchea@univ-lorraine.fr; 6 Marine Ecosystems and Organisms Research Laboratory (MERSEA), University of Caen Normandy, Esplanade de la Paix, 14032 Caen, France

**Keywords:** early life history stages, evo-devo, ontophylogenesis, ontogeny, phylogeny, three item analysis

## Abstract

It has been hypothesized that a lack of dioxygen could be the trigger of hatching. Here we address the question of whether developmental heterochronies could be an indirect way to study adaptation to lack of dioxygen. To answer this question, we aim to explore the potential of an original method which compares the relative rate of anatomical development across an extended set of 19 species using a hierarchical analysis of ontogenetic time. Then we compare heterochronic rates with the relative time at hatching and information about dioxygen availability. Pairwise independence between dioxygen richness, rate of development and relative time at hatching are all rejected at the 5% threshold. This suggests a link between these three variables, and provides consilient evidence for the hypothesis that lack of dioxygen is a trigger of hatching. A possible scenario is the following: hatching early relatively to developmental time (because of a lack of dioxygen) exposes larvae to predation, and concomitant accelerated early anatomical development could be the result of a selective constraint which consists of being able to escape predators. We conjecture that accelerated embryonic development might be linked to adaptation against predation in dioxygen-poor waters.

## 1. Introduction

Developmental heterochrony is an important process by which new anatomical or morphological characters [1,2,3,4] and possibly adaptations [5,6,7] appear during the course of evolution. Heterochrony can be defined as a change in the timing or rate of development relative to the ancestor [8]. Since the late 1970s, the phenomenon has been largely studied through the relationship of size and shape, what Smith [3] called “growth heterochrony”. For example, heterochrony explains the diversity of jaw morphology within the family of needlefishes (Belonidae) from halfbeak to needlenose [9]. Considering a developmental trajectory as a series of discrete events, a “sequence heterochrony” [3] describes the change in a sequence position of an event relative to others. For example, Fuiman [10] observed that some morphological features appeared earlier during the development of flatfishes compared to pelagic species, especially for the sensory, locomotor and digestive systems. Fuiman [11] tried to construct a phylogeny of the teleostean family Catostomidae based on larval traits (including relative timing of developmental events) and found that (1) the phylogeny of species based on larval characters failed to recover some of the groups found based on adult characters and (2) several characters showed a phylogenetic acceleration in development. Shardo [12] showed that the American shad *Alosa sapidissima* displayed delayed hatching, neuromasts, and opercular development relative to other clupeoids. Each time the change made to the timing or to the rate of development of a character or a set of characters is plausibly driven by natural selection, heterochrony has something to do with adaptation [2,5,8,13]. Examples have been described several times, such as for salamanders [5], spatangoid echinoids [6], humans [7] and sequence heterochronies, and have even been simulated to show their adaptive role in the evolution of neural networks [14,15].

Teleostean fishes exhibit a remarkable diversity of reproductive traits [16,17,18,19]. Species do hatch at different times in relation to their developmental stage [20]. Therefore, what is called “hatching”—a landmark event—occurs at strongly different degrees of development for organs in teleosteans [18,21,22]. Hatching not only occur at different degrees of morphological development between teleostean fish species but also at different moments compared to the total duration of the early life stage period. We call this phenomenon “relative hatching time”, contrasting with the absolute hatching time (normalized to temperature and measured with Celsius degrees.hours). Moreover, independently of their absolute hatching time, fish species exhibit heterogeneous rates of embryo–larval change during the course of their own development and across species: some accelerate at the beginning, and others accelerate at the end. For instance, in a developmental time frame (normalized to temperature) from fecundation to the appearance of lepidotricha, huchen (*Hucho hucho*) is late at the beginning of its development relative to other species while tench (*Tinca tinca*) accelerates at the start of its development compared to others [23]. As a result, there is a complex relationship between absolute hatching time and the hatching form across species, a situation which contributes to the inconsistency of the concept of larva [23,24,25,26,27]. For instance, mouth opening is achieved before hatching in medaka (*Oryzias latipes*) and after hatching in Atlantic cod (*Gadus morhua*). Similarly, eye pigmentation starts before hatching in stone moroko (*Pseudorasbora parva*) and after hatching in silver barb (*Barbonymus gonionotus*). This means that Balon [25], Kendall et al. [28] and Aral et al. [29] do not segment developmental stages of teleosteans in the same way, limiting a true comparison of early life stages between fish species. As developed above, hatching is an obvious landmark to describe early life stages, but a comparative approach forces us to acknowledge that it is not (solely) a matter of degree of anatomical development. It has been hypothesized that lack of dioxygen could be the trigger of hatching [30]. As heterochronies help to understand adaptations, we address the question of whether fish developmental heterochronies could be an indirect way to study adaptation to lack of dioxygen. To answer this question, we aim to explore the potential of the method of Lecointre et al. [23]. Indeed, as heterochrony is a key process that generates a large variety of forms and life-history traits, it requires an objective method to be properly assessed.

A semaphoront [31,32] is an individual organism at a specific point in its development or life cycle. Lecointre et al. [23] developed a method to compare the tempo of embryo–larval development among fish species by mixing semaphoronts of different developmental moments from different species within a single hierarchical graph (a “tree”). They showed that this comparative approach was suitable to detect the phenomenon of heterochrony.

**Figure 1:**
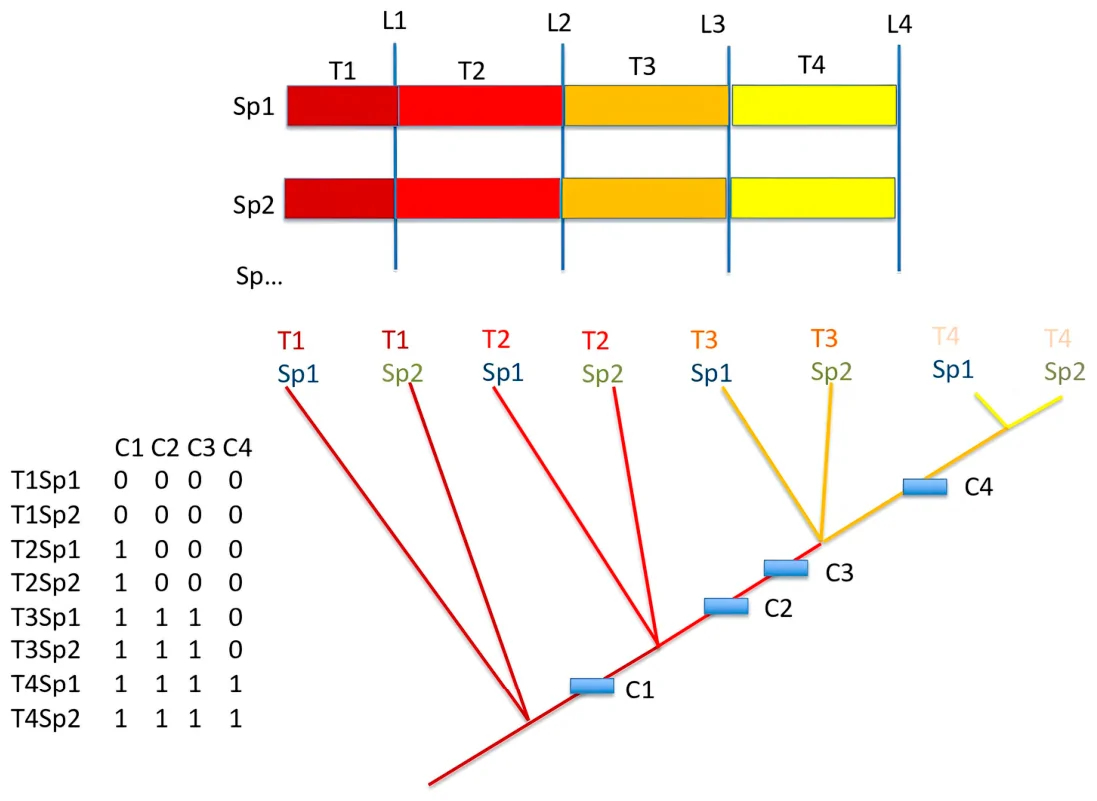
Theoretical framework: Sp1, sp2, etc.: species; T1, T2, etc.: arbitrary time periods; L1, L2, etc.: landmarks. L4 closes the developmental time with 100% achievement, arbitrarily set as the appearance of lepidotricha in fins by Lecointre et al. [23], set as the start of exogeneous feeding here. On the left, an example of the data matrix is given. See text.

**Figure 2:**
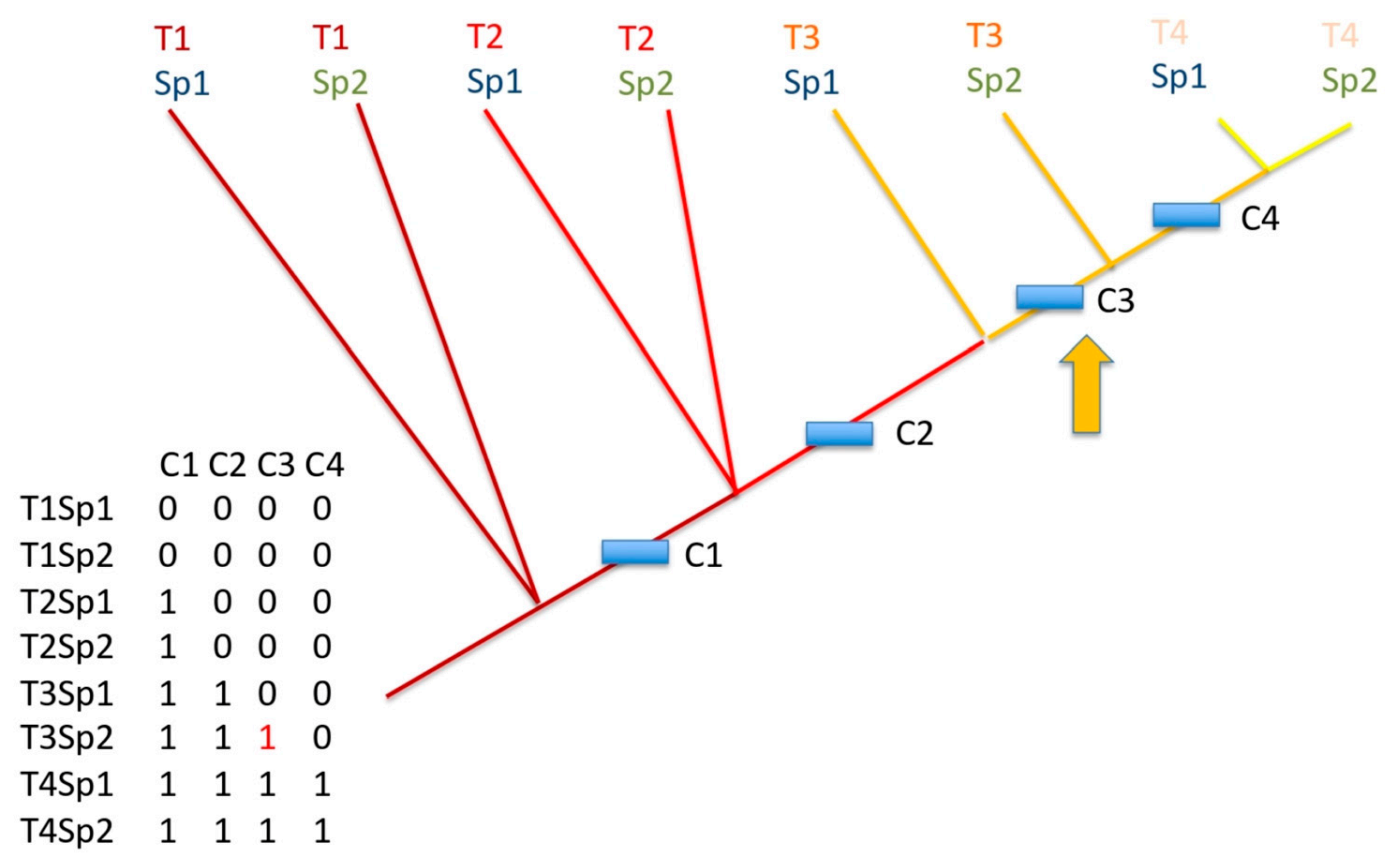
Time hierarchy (right) from the matrix on the left, showing that at time window 3, species 2 is advanced compared to species 1 because it already has character 3. Otherwise, species 1 is late compared to species 2. Such a hierarchy of ontogenetic time allows detection of heterochronies [23].

Semaphoronts are defined for all species according to arbitrary time windows (Figure 1, top) along with the complete developmental time measured in degree-hours of development (temperature*time, with temperature in degrees Celsius (°C) and time in hours). As the absolute developmental time depends on temperature, the developmental time is normalized by temperature to allow comparisons across species. The starting point of the method is a matrix (Figure 1, left) where columns are characters (presence of organs and traits) and rows represent a given species at a given time window (a semaphoront *sensu* Hennig, [31,32]), constructed as follows. From fecundation to juveniles, the time (measured in Celsius degrees.hours) of first appearance for each of the chosen anatomical structures (Figure 1, left: C1, C2, C3, etc.) is recorded for each species. The total embryo–larval developmental time is arbitrarily segmented as a % (e.g., 25%, 50%, 75% as shown at the top of Figure 1). Then the first appearance of each structure can be assigned to a time segment. Each species (Sp1, Sp2…) at a given time segment is a semaphoront. For each species, each anatomical trait of interest is coded “0” if absent at the corresponding time segment and “1” if present, in the data matrix. A cladistic analysis produces a rooted non-cyclic connected graph to hierarchize the ontogenetic time (Figure 1, right). This hierarchy allows us to formally detect heterochronies. For instance, if species 1 at time segment 3 does not have the organ C3 (Figure 2), the tree will exhibit a heterochrony: species 1 is late at time 3. No reference phylogeny is necessary for the method; the only requirement is the data on the presence or absence of a given organ in a given taxon at a given time window. The approach generates a tree showing a time hierarchy of semaphoronts, allowing developmental heterochronies to be objectively detected. The tree is also suitable for naming ontogenetic stages for several species, at least potentially. By doing so the terminology attains a wider domain of validity, but this issue is not in the scope of the present work. Lecointre et al. showed that the method is robust, i.e., the results do not depend on the arbitrary landmarks chosen to define time windows [23].

Much effort has been previously made to understand and analyze developmental characters and developmental sequences within a phylogenetic framework [3,13,33,34,35,36,37] and subsequent studies have focused on the detection of heterochronies and the phylogenetic usefulness of developmental characters. However, no study has mixed semaphoronts of different stages from different species within a single tree, without any preconception of developmental stage (e.g., larva, juvenile, adult, etc.). Lecointre et al. [23] showed the usefulness of this approach to detect heterochronies and to allow a new type of staging, precisely because the method does not start from reconceived stages, but rather from time segments. Last but not the least, the hierarchy we use here to detect heterochronies is not a species phylogeny, but a time hierarchy (see below).

Using a hierarchical analysis of ontogenetic time, we explore the relationships between the relative time of hatching and the relative rate of anatomical development across an extended set of 19 species through a three-item analysis for cladogram construction. Then we compare the detected heterochronies and relative hatching times with data on dioxygen availability to explore whether conjectures can be made concerning the role of dioxygen availability on hatching.

## 2. Materials and Methods

### 2.1. Sampling

The development duration is set from fecundation (0%) to the start of exogeneous feeding (time set at 100%). This 100% boundary is chosen because it is late enough to cover a large duration of development and it is present in all descriptions. Between this, we arbitrarily segmented developmental time (in Celsius degrees.hours) into six periods: 0–5%, 5–10%, 10–25%, 25–50%, 50–75%, and 75–100%.

Species (Table 1) and traits (Table 2) were chosen to summarize present knowledge available from full developmental tables that were previously published and are comparable to each other. Details about conditions of organism observations are provided within each article cited in Table 1. This resulted in 19 species. Characters were observed and data completed for only one of them, the Orchid dottyback (*Pseudochromis fridmani*). Live embryos were provided by a commercial supplier, Recifathome (the address is provided on the title page). No “experiments” were made on these live embryos, but direct observation of the developing fish before the stage of autonomous feeding was performed at Recifathome. In order to complete previous knowledge, these non-invasive observations consisted of recording the onset of structures in the developing embryo via transparency under a Olympus SZ61 binocular (Olympus France S.A., Rungis, France), coupled with a camera Olympus SP23 (Olympus France S.A., Rungis, France). Three individuals were observed for each data point. As observations were restricted to early stages prior to free-feeding, ethical approval was not required according to European Union regulations on animal research. As shown in Table 1, a large array of families from diverse geographical origins (Europe, Asia, Africa, Americas) was studied, with species from diverse environments and temperature preferences, diverse trophic regimes and economic interest (aquaculture, fishing, ornament). Traits (or characters to code) are embryo–larval structures that can be observed using a binocular microscope; they are the same as Lecointre et al. [23] with slight modifications to adjust the availability of data for the 19 species. The coded presence/absence of traits (or structures) (columns in Table 3) for each semaphoront (lines in Table 3) is given in Table 3.

### 2.2. Tree Search

As most organs/traits appear in a cumulative manner, the nested hierarchy provided by a non-cyclical-oriented connected graph is suitable for ordering the onset of organs development. Such graphs are usually called “rooted trees”. At each node of these trees, it is possible to identify which organs appear. Moreover, as our method is ultimately useful for proposing a taxonomy of developmental stages made up of nested categories that best reflect the differentiation process of ontogenetic development, we chose an analytical approach consistent with a hierarchical way of thinking. In his work *Über Entwickelungsgeschichte der Thiere. Beobachtung und Reflexion* (translated into English in 1853 by Thomas Henry Huxley in his *Scientific Memoirs*), Karl Ernst von Baer [70] made fundamental statements about his hierarchical view of comparative embryology, and these were summarized into four points by Huxley: (1) the more general characters of a large group appear earlier in the embryo than the more special characters; (2) after the most general forms appear, the less general are developed, and so on, until finally the most special develop; (3) every embryo of a given animal form, instead of passing through other forms, is separated from them; (4) the embryo of a higher form never resembles any other form, only its embryo. Point 1 reflects time hierarchy, point 2 is differentiation, point 3 is divergence, and point 4 is anti-recapitulation: a developmental stage of a more complex species never represents the adult stage of a less complex species; it only looks like an embryonic stage of a less complex species.

There are two consequences of this: first, there is no “recapitulation” in the “tree” produced here. It is not a species phylogeny. In a phylogenetic reconstruction of species relationships, the distribution of character states is supposed to partially vary among species, susceptible to a phylogenetic hierarchy of character state distribution. Here, character states (availability of a given structure) are the same across all species. The hierarchy provided is a time hierarchy, not a phylogenetic one. As a result, the relationships of semaphoronts within the same time segment do not reflect species phylogenetic relationships.

Second, the method called “three item analysis” (3ia hereafter [71]) considers characters as hierarchies where character states are nested within each other. Passing from one state to another (0 to 1) is not a “transformation” but a “differentiation”, a concept which is suitable for the study of development, as shown by von Baer [70] (0 does not “transform” to 1, but rather 1 is a modified version of 0, [71,72], as indicated by point 2 above). As development is a cumulative process through time, the hierarchy provided by the tree is a time hierarchy (point 1 above). Therefore, it is logical to define the outgroup (the root of the tree) at the boundary 0%: outgroups are semaphoronts at 0% of their development (no traits). The 3ia analysis was conducted using the software AGATTA version 0.9.1 (2025) [73]. The original hierarchical characters are decomposed into triplets, i.e., minimal relational statements. Each triplet is weighted through the procedure of fractional weighting [74,75]. Then, AGATTA is coupled with the wQFM [76] version 1.4 (April 2022) heuristic to produce an optimal tree that best represents the original triplet set. wQFM is designed to produce dichotomic trees only; then, we collapsed nodes without triplet support by hand. Unambiguous synapomorphies were extracted from the synapomorphy test performed by the --*chartest* option of AGATTA, and the Retention Index [77,78] for 3ia [79,80] was extracted from the --*ri* request of AGATTA.

## 3. Results

**Figure 3:**
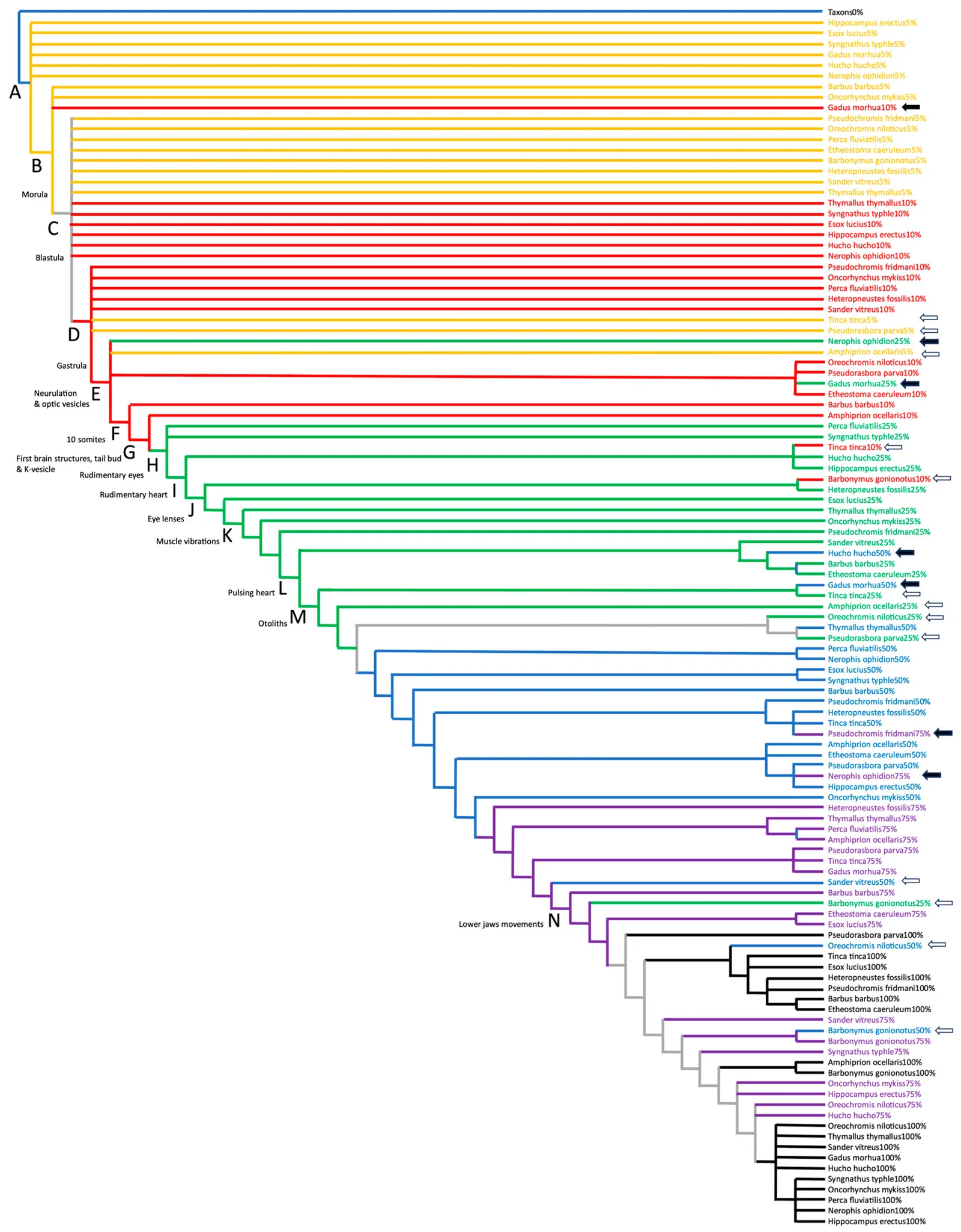
Optimal tree retained: colors are associated with pre-defined time segments: Yellow: 0% to 5%; red: 5% to 10%; green: 10% to 25%; blue: 25% to 50%; purple: 50% to 75%; black: 75% to 100%; grey: undetermined. Black arrows indicate semaphotonts that are late compared to other time segments; white arrows indicate semaphoronts that are advanced compared to other time segments. Clades with unambiguous synapomorphic traits are indicated with a letter, and traits that passed the synapomorphy test through the chartest option of AGATTA are indicated. The Retention Index is 0.98.

AGATTA generated 236,517 triplets. Figure 3 shows the optimal tree from the triplets retained, constructed from combined quartets using wQFM, with colors associated with the pre-defined time segments. The Retention Index is 0.98, showing a high degree of nested hierarchy in the data. Clades A, B, C, D, and E exhibit a number of collapsed branches, which is due to a synchronic rise in traits across species under the time segments considered. However, a number of cross-species heterochronies was observed.

### 3.1. Late Species

*Gadus morhua* is late at 10% (not included in node C), 25% (not included in node H) and 50% (grouped with other species at 25%). At 10%, *Gadus morhua* has not yet reached the blastula stage. At 25%, it has not yet developed the 10 somites, first brain structures, the tail bud, Kupffer vesicle or rudimentary eyes that other species have at this time. *Nerophis ophidion* is late at 25% (not yet a member of node H) and 75% (grouped with other species at 50%). At 25%, it has not yet developed the 10 somites, first brain structures, the tail bud, kuppfler vesicle or rudimentary eyes that other species have at this time. *Hucho hucho* is late at 50% (grouped with other species at 25%). *Pseudochromis fridmani* is late at 75% (grouped with other species at 50%).

### 3.2. Species in Advance That Hatch Early

*Barbonymus gonionotus* is particularly advanced at 10% (a member of clade J) and 25% (a member of clade N) and is still advanced at 50%, close to other species at 75% and 100%. At 10%, it already has a rudimentary heart and eye lenses, which other species do not have at this stage. At 25% it already performs lower jaw movements that usually appear at 75% for other species (hatching at 17%).

*Tinca tinca* is advanced at 5% (a member of clade D), 10% (a member of clade I), and 25% (a member of clade M), i.e., all along the first quarter of its embryonic development (hatching at 30%). At 5%, gastrulation is already achieved; at 10% it already has a rudimentary heart and eyes, which other species do not have at this stage. At 25% it already has otoliths, which most other species do not have at this point (except species with accelerated developments).

*Oreochromis niloticus* is advanced at 25% (a member of clade M) and 50% (branched with other species at 100%) (hatching takes place at 32%). At 25% it already has otoliths, which most other species do not have at this stage (except species with accelerated developments).

### 3.3. Two Species Have Rapid Early Developments While Hatching Late

*Pseudorasbora parva* is advanced at 5% (a member of clade D) and 25% (a member of clade M, with hatching at 60%). At 5%, gastrulation is already achieved. At 25% it already has otoliths, which most other species do not have at this stage (except species with accelerated developments), and is closer to the 50% stage of other species.

*Amphiprion ocellaris* is advanced at 5% (a member of clades D and E), slightly advanced at 10% (a member of clade G), and still advanced at 25% (a member of clade M, with hatching at 69%). At 5% it has already undergone gastrulation and neurulation while other species have not; at 10% it already has brain structures, a tail bud and Kupffer’s vesicle, which most other species do not have at this stage, and at 25% it already has otoliths, which most of other species do not have at this stage (except species with accelerated developments).

*Sander vitreus* is slightly advanced at 50%, branched alongside other species at 75% (with hatching at 43%).

## 4. Discussion

Such a hierarchy of ontogenetic time as that presented here has theoretical, epistemological and biological issues. The theoretical issues with regard to the historical program of the discipline called “Evo-Devo” are discussed elsewhere [81,82,83]. The epistemological issues are the possibility to detect cross-species heterochronic developments [23] and the potential to establish a nomenclature of developmental stages under the form of nested sets [23,84], with each stage being based on anatomical character. The biological issues are multifold, including the link between heterochronies and environmental factors and selective constraints. This is the one we propose to discuss here, especially with regard to the availability of dissolved dioxygen during embryo–larval development.

### 4.1. Comparing Relative Hatching Time with Dioxygen Availability

Teletchea and Pauly [30] developed the hypothesis that hatching occurs when dissolved oxygen acquired by diffusion through the egg surface is no longer sufficient to cope with the needs of the developing embryo: “*When the body surface of the embryo is about 1.5 that of the egg surface, the lack of oxygen that the embryo suffers triggers both the secretion of chorionase and the activity of embryo (Keckeis et al. 1996; Czerkies et al. 2001; Fuiman 2002; Kamler 2002)”* [22,85,86,87]. Considering species that undergo an accelerated early anatomical development, and those which are late in their development, our results seem to corroborate the hypothesis.

**Figure 4:**
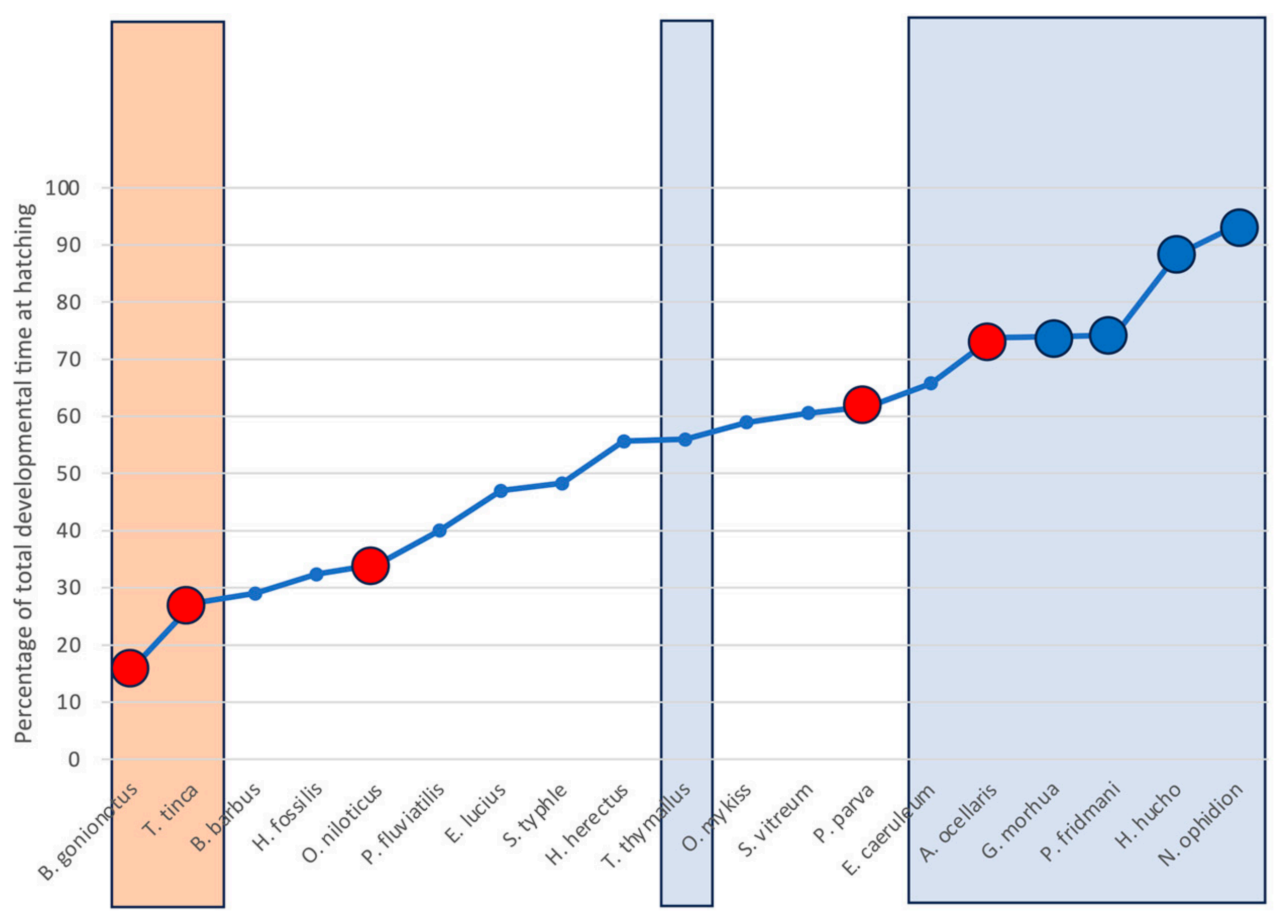
The ratio of the incubation time and the time duration of development to reach the 100% stage (with time normalized to temperature). On the left, species that hatch early are presented, on the right those that hatch late are presented. The orange area represents poor dioxygen conditions, and the blue area represents dioxygen-rich conditions, either physically (*Hucho hucho*, *Gadus morhua*, *Etheostoma caeruleum*, *Thymallus Thymallus*) or through parental behavior (*Nerophis ophidion*, *Pseudochromis fridmani*, *Amphiprion ocellaris*). Blue circles are species that have delayed anatomical development compared to other species (Figure 3). Red circles are species with accelerated anatomical development compared to other species (Figure 3).

Species are ranked (Figure 4) according to the ratio between temperature-normalized incubation time and temperature-normalized full development time to reach the 100% boundary, i.e., the percentage of developmental time at hatching. On the left are early hatching species (*Barbonymus gonionotus*: 15%) and on the right the late hatching species are presented (*Nerophis ophidion*: 92%). The former undergoes most of its development outside the eggshell while the later undergoes most of its development inside it. The relationship between percentage of developmental time at hatching and the optimal developmental temperature for each species was controlled and exhibited no relation (R^2^ = 0.245).

The four species that are late at hatching in their development compared to others (*Nerophis ophidion*, *Hucho hucho*, *Pseudochromis fridmani*, and *Gadus morhua*) are all on the right side, i.e., above 66% (the last third) of their total embryo–larval development. These species seem to have time to develop without constraint. Temperature has nothing to do with this, as shown in our study where developmental time is normalized by temperature. Indeed, we find on the right “cold species” (*Gadus morhua* laying in January in Northern Atlantic seawaters, at 3 °C), “temperate species” (*Nerophis ophidion*) as well as “tropical species” (*Pseudochromis fridmani*, see Table 1). Similarly, being either a marine (e.g., cod) or freshwater (e.g., huchen) fish does not seem to be a criterion for late hatching. Five species hatch early, i.e., before the first third of their development (*Barbonymus gonionotus*, *Tinca tinca*, *Barbus barbus*, *Heteropneustes fossilis* and *Oreochromis niloticus*, all of which are freshwater species). Last but not the least, the two species which exhibit the most accelerated early development (Figure 3) hatch the earliest, before 30% of their relative developmental time: *Barbonymus gonionotus* and *Tinca tinca*. This suggests a constraint that triggers early hatching, and early accelerated development as a selective consequence of this constraint. This will be discussed over the next few sections.

### 4.2. Accelerated Embryo–Larval Anatomical Developments and Dioxygen Availability

Teletchea and Pauly [30] suggested that this constraint could be due to dioxygen availability, in line with the observations of Dhaneesh et al. [40] who noticed the following while rearing *Amphiprion percula*: “*the developmental rate of fertilized eggs profoundly varied with temperature and also with oxygen content of water*”. It is therefore interesting to compare both relative hatching time and acceleration of anatomical development with dioxygen availability. *Barbonymus gonionotus* lay in dormant, organic-rich freshwaters of flooded plains of South-east Asia [42] at temperatures between 25 °C and 33 °C [43], suggesting poorly oxygenated waters. *Tinca tinca*, a species known for its tolerance to poor dioxygen conditions [61, 67] lay between April and September (depending on the area) in bottom still waters of lakes loaded with organic matter; numerous green eggs stuck on plants or directly on the bottom between 12.5 °C and 25.6 °C [68], most often at about 19 °C to 20 °C. *Pseudorasbora parva* advances until 25% of its development, which is consistent with the fact that breeding occurs in habitats with still or very slow-flowing water [45], with the possibility of limited dioxygen access. An apparent paradox is that hatching occurs at 62% of its developmental time. It is difficult to link this to the fact that parents guard and clean the eggs [45,65], and the sister-group of this species, *Pseudorasbora interrupta* [88], should be investigated further in order to estimate phylogenetic inertia in the life-history traits of the two species.

Following the idea that hatching is triggered by a lack of dioxygen [30], we suggest that hatching early relative to a species’ developmental time could be mainly due to embryonic anoxia and exposure of free young larvae to predation. Concomitant accelerated anatomical development could be the result of a selective constraint on growth and locomotion abilities which correlates with being able to escape predators. Accelerated embryonic development could therefore be linked to an adaptation against predation in dioxygen-poor waters.

### 4.3. Delayed Embryo–Larval Anatomical Developments

On the other hand, it is also interesting to compare relative hatching time with delayed anatomical development and dioxygen availability. Species on the right develop in oxygen-rich environments. *Hucho hucho* lay their eggs upstream in gravel, slightly buried up to 40 cm, in flowing waters that are fresh (6–9 °C) and well-oxygenated (the same situation occurs for *Etheostoma caeruleum*, but in warmer conditions: 17–19 °C [48,49]). *Gadus morhua* lay between January and April in the northern Atlantic in cold (3 °C, and then oxygenated) waters. Interestingly, the tropical *Pseudochromis fridmani* lays eggs at 27 °C in less oxygenated waters, but parents actively ventilate their eggs [61]. Consequently, these eggs do not suffer early from lack of dioxygen and most of the development is achieved inside the egg. The same interesting phenomenon is noticeable in syngnathids. Among the three syngnathid species sampled here, *Nerophis ophidion* is the only one that has its eggs stuck to the male trunk (not enclosed in a pouch), making it better exposed to dioxygen. Last but not the least, males transporting eggs change their behavior, and swim closer to the surface [56]. Studying four species of synthathids, including *Entelurus aequoreus* and *Nerophis ophidion* who perform simple ventral attachment of eggs onto the male trunk, and *Syngnathus typhle* and *S. rostellatus* with fully enclosed brood pouches on the tail, Braga Goncalves et al. [56] showed that under experimental hypoxia, all species showed lower embryo survival, while species with brood pouches suffered greater embryo mortality compared to pouchless species, irrespective of oxygen treatment. The authors recorded that species without pouches spend more time closer to the surface, possibly to improve oxygenation. Here we found that *Nerophis ophidion* hatches very late and is late in its development compared to the two other syngnathid species which have pouches.

### 4.4. A Possible Link Between Dioxygen, Developmental Rate and Relative Hatching Time

Three categories were defined with regard to dioxygen availability: a dioxygen-poor environment (orange zone in Figure 4), medium dioxygen content (white zone in Figure 4), and dioxygen-rich environment (blue zone in Figure 4); three categories were defined with regard to the rate of development: accelerated early development (red circles), a "normal" rate, and delayed development (blue circles); three categories were defined with regard to hatching time: hatching in the first third of total development time (0–33%), hatching in the second third of total development time (33–66%), and hatching in the last third of total development time (66–100%). The 19 species were distributed throughout these categories (see Appendix A), and chi-square pairwise independence tests were performed (degree of freedom of 4).

The null hypothesis of independence between the three categories of dioxygen availability and three categories of rate of development is rejected at the 5% threshold (with an expected chi-square value of 9.488 and observed value of 14.766; the *p*-value is 0.0052). Similarly, independence between the three categories of hatching time and three categories of rate of development is rejected at the 5% threshold (with an expected chi-square value of 9.488 and observed value of 15.089; the *p*-value is 0.0045). Finally, independence between the three categories of dioxygen availability and three categories of hatching time is rejected at the 5% threshold (with an expected chi-square value of 9.488 and observed value of 20.283; the *p*-value is 0.0004).

The medium values of dioxygen content could partly be artifactual due to incomplete information. To check for the impact of these possible shortcomings, we performed the test with the two extreme categories only: a dioxygen-poor environment (orange zone) and oxygen-rich environment (blue zone) for dioxygen content; accelerated early development (red circles) and delayed development (blue circles) for rate of development; and hatching in the first third of total development time (0–33%) and hatching in the last third of total development time (66–100%), generating one degree of freedom for each pairwise test. Independence between the two categories of dioxygen availability and two categories of rate of development cannot be rejected at the 5% threshold (with an expected chi-square value of 3.841 and observed value of 3.733; the *p*-value is 0.0533). Independence between the two categories of hatching time and two categories of rate of development is rejected at the 5% threshold (with an expected chi-square value of 3.841 and observed value of 4.8; the *p*-value is 0.0285). Independence between the two categories of dioxygen availability and two categories of hatching time is rejected at the 5% threshold (with an expected chi-square value of 3.841 and observed value of eight; the *p*-value is 0.0047).

Overall, independence between categories is rejected; thus, there must be a link between categories of dioxygen availability, relative hatching time, and rate of organism development. Obviously, a chi-square test is not a cause–effect demonstration, but allows a relationship to be suspected between the three variables. We do not claim that we are proving the hypoxic hypothesis in the experimental sense. Here, this hypothesis is reinforced via additive consilience. Gray [89] identifies and explains “*key reasoning strategies such as retrodiction, abduction, consilience of evidence, and model-based inference*” and describes “*how they operate in conjunction with scientific practices*”. Abductive reasoning is “*selecting the most plausible explanation from incomplete evidence*”, while consilience of evidence is *“integrating independent lines of data*”, and model-based inference is “*constructing theoretical representations to bridge evidence and explanation*”. This study contributes consilient information to the hypothesis of Teletchea and Pauly [30] in that lack of dioxygen is a trigger of hatching.

### 4.5. Limitations

As the tree has a strongly pectinate structure, this indicates that the ontogenetic differentiation of the teleost stages in our sample can be adequately represented by a single series of successive differentiations. However, there are a few cases where the presence of symmetric nodes suggests that it is impossible to represent the changes as a single series, and therefore, to make comprehensive comparisons. In particular, the sister clade of *Pseudorasbora parva* at 100% is a symmetric node from which two incomparable series of differentiations diverge. In this case, it is possible, for example, to say that *Oreochromis niloticus* at 50% is ahead of several 100% stages (*Esox*, *Tinca*, etc.), but not all of them (*Thymallus*, *Sander*, *Gadus*, etc.).

The present method allows the comparison of dozens of species; however, the number of semaphoronts compared should be multiplied by the number of time windows. This generates a number of terminals (number of “taxa”) that grows much faster than the number of characters we were able to observe, leading to potential problems of resolution in the hierarchy (which we did not encounter here).

Precise field data about dioxygen content of natural waters where embryos develop are difficult to obtain and collect for a vast array of species with diverse ecological requirements. Further investigations would be significantly improved by such data.

### 4.6. Further Questions

There are open questions still to be investigated. We do not claim that the lack of dioxygen availability is the only factor explaining accelerated early developments, as other selective constraints could be at play. For instance, *Amphiprion ocellaris* is advanced until 25% of its development and nevertheless this species hatches late (at 72% of its developmental time); this could be associated with dioxygen availability (blue area Figure 4) due to the active role of parents who ventilate the eggs [40]. Accelerated development could be a form of phylogenetic heritage that persists disregarding dioxygen availability. However, in the sister-group of *Amphiprion ocellatus*, namely *Amphiprion percula* [90,91,92,93], ventilation of eggs by males is also observed [94,95], rendering this conjecture unfruitful. Dioxygen availability is not always associated with delayed anatomical development: while the spawn of *Etheostoma caeruleum* and *Thymallus Thymallus* in well-oxygenated freshwaters [64] (blue area Figure 4) do hatch late (67% and 57% respectively), they do not exhibit noticeable late anatomical development compared to the other species. This is not really a contradiction, as it can simply reflect an absence of constraint. A likely constraint in accelerated early anatomical development is a lack of dioxygen, not its availability, which allows, but not forces, delayed anatomical development.

## 5. Conclusions

Developmental heterochronies provide potential mechanisms for adaptation. Here the hierarchical analysis of ontogenetic time allows us to detect species with accelerated early development and species with delayed development. When comparing them with dioxygen availability and relative developmental time at hatching, it appears that pairwise independence between dioxygen richness, rate of development and relative time at hatching are all rejected at the 5% threshold. This suggests a link between these three variables, bringing consilient information to the hypothesis that a lack of dioxygen is a trigger of hatching. A possible scenario is the following: hatching early relative to a species’ developmental time (because of a lack of dioxygen) exposes larvae to predation, and concomitant accelerated anatomical development could be the result of a selective constraint which consists of acquiring sensory and locomotor systems early enough to be able to escape predators after hatching. We make the conjecture (for further research) that accelerated embryonic development could be linked to an adaptation against predation in dioxygen-poor waters.

## Figures and Tables

**Table 1:** Species list and reference for developmental description (third column).

Scientific Name	Family	Ref.	Climate	Water	Environment	Temp. Range	O_2_-Rich	O_2_-Poor	Neither
*Amphiprion ocellaris*	Pomacentridae	[38,39]	Tropical	Marine	Recif-associated	23 to 28	Ventilation [40]		
*Barbonymus gonionotus*	Cyprinidae	[41]	Tropical	Still freshwater	Benthopelagic, potamodromous	22 to 28		[42,43]	
*Barbus barbus*	Cyprinidae	[44]	Temperate	Flowing freshwater	Benthopelagic, potamodromous	10 to 24			[45]
*Esox lucius*	Esocidae	[46]	Temperate	Still freshwater	Brackish, pelagic, potamodromous	10 to 28			[45]
*Etheostoma caeruleum*	Percidae	[47]	Temperate	Flowing freshwater	Benthopelagic	4 to 18	Environment [48,49]		
*Gadus morhua*	Gadidae	[50]	Cold temperate	Marine	Brackish, oceanodromous	0 to 15	Environment [51]		
*Heteropneustes fossilis*	Heteropneustidae	[52]	Tropical	Still freshwater	Brackish, demersal	21 to 25			[53]
*Hippocampus erectus*	Syngnathidae	[54]	Subtropical	Marine	Recif-associated	10 to 27			[53]
*Hucho hucho*	Salmonidae	[55]	Temperate	Flowing freshwater	Benthopelagic, potamodromous	6 to 18	Environment [45]		
*Nerophis ophidion*	Syngnathidae	[54]	Temperate	Marine	Brackish, demersal	8.6 to 19	Behavior [56]		
*Oncorhynchus mykiss*	Salmonidae	[57]	Temperate	Anadromous	Brackish, benthopelagic	10 to 24			[45]
*Oreochromis niloticus*	Cichlidae	[58]	Tropical	Still freshwater	Brackish, benthopelagic, potamodromous	13.5 to 33			[53]
*Perca fluviatilis*	Percidae	[59]	Temperate	Still freshwater	Brackish, demersal	10 to 22			[45]
*Pseudochromis fridmani*	Pseudochromidae	[60], this study	Tropical	Marine	Recif-associated	23 to 28	Ventilation [61]		
*Pseudorasbora parva*	Cyprinidae	[62]	Temperate	Still freshwater	Brackish, benthopelagic	5 to 22			[45]
*Sander vitreus*	Percidae	[63]	Temperate	Still freshwater	Brackish, benthopelagic, potamodromous	1 to 29			[53]
*Syngnathus typhle*	Syngnathidae	[54]	Temperate	Marine	Brackish, demersal	8 to 24			[53]
*Thymallus thymallus*	Thymallidae	[64]	Temperate	Flowing freshwater	Brackish, benthopelagic	6 to 18	Environment [45,65]		
*Tinca tinca*	Tincidae	[66,67]	Temperate	Still freshwater	Brackish, demersal, potamodromous	4 to 24		[45,68]	

**Table 2:** List of 41 traits (from Lecointre et al. [23], slightly modified; these characters are described in the work of Kunz [69]).

1	Fertilization	26	First pigmentation on the body
2	Perivitelline space	27	Lower jaw starts moving
3	Blastodisc	28	Gill lamellae begin development
4	Cleavage	29	First movements of pectoral fins
5	Two blastomeres	30	Mouth opening
6	Four blastomeres	31	Start of exogenous feeding
7	Morula	32	Yolk has completely disappeared
8	Blastula	33	Lepidotrichia in caudal fin bud
9	Gastrula	34	Lepidotrichia in dorsal fin bud
10	Neurulation	35	Lepidotrichia in anal fin bud
11	Somites (around 10)	36	Lepidotrichia in pelvic fin bud
12	Rudimentary heart	37	Kupffer’s vesicle apparition
13	Rudimentary eyes	38	Otic vesicle = auditory vesicle
14	First brain structures	39	Blood circulation
15	Tail bud	40	Optic vesicles
16	Eye lenses	41	First melanophore
17	Muscle vibrations		
18	Full number of somites		
19	Pulsating heart		
20	Embryonic finfold begins		
21	First hatching		
22	Rudiment of the pectoral fins		
23	Otoliths appear		
24	Last hatching		
25	Pigment of the eye		

**Table 3:** Data matrix: A column is a character (absence (0)/presence (1)); the first column represents the first trait in Table 2 and the 41st column represents the 41st trait in Table 2; and a line is a semaphoront, i.e., a species in Table 1 at a particular period of its developmental time.

	1	2	3	4	5	6	7	8	9	10	11	12	13	14	15	16	17	18	19	20	21	22	23	24	25	26	27	28	29	30	31	32	33	34	35	36	37	38	39	40	41
Taxons 0%	0	0	0	0	0	0	0	0	0	0	0	0	0	0	0	0	0	0	0	0	0	0	0	0	0	0	0	0	0	0	0	0	0	0	0	0	0	0	0	0	0
*A. ocellaris* 5%	1	1	1	1	1	1	1	1	1	?	0	0	0	0	0	0	0	0	?	0	0	0	?	0	0	0	?	0	?	0	0	0	0	0	0	0	0	0	0	?	0
*A. ocellaris* 10%	1	1	1	1	1	1	1	1	1	?	0	0	0	1	1	0	0	0	?	0	0	0	?	0	0	0	?	0	?	0	0	0	0	0	0	0	1	0	0	?	0
*A. ocellaris* 25%	1	1	1	1	1	1	1	1	1	?	1	1	1	1	1	1	1	1	?	0	0	0	?	0	1	1	?	0	?	0	0	0	0	0	0	0	1	1	0	?	1
*A. ocellaris* 50%	1	1	1	1	1	1	1	1	1	?	1	1	1	1	1	1	1	1	?	0	0	1	?	0	1	1	?	0	?	1	0	0	0	0	0	0	1	1	1	?	1
*A. ocellaris* 75%	1	1	1	1	1	1	1	1	1	?	1	1	1	1	1	1	1	1	?	0	1	1	?	0	1	1	?	1	?	1	0	0	0	0	0	0	1	1	1	?	1
*A. ocellaris* 100%	1	1	1	1	1	1	1	1	1	?	1	1	1	1	1	1	1	1	?	1	1	1	?	1	1	1	?	1	?	1	1	1	1	0	0	1	1	1	1	?	1
*S. vitreus* 5%	1	1	1	1	1	1	1	1	0	0	0	0	0	0	0	0	0	0	0	0	0	0	0	0	0	0	0	?	0	0	0	?	0	?	?	?	0	0	0	0	0
*S. vitreus* 10%	1	1	1	1	1	1	1	1	1	0	0	0	0	0	0	0	0	0	0	0	0	0	0	0	0	0	0	?	0	0	0	?	0	?	?	?	0	0	0	0	0
*S. vitreus* 25%	1	1	1	1	1	1	1	1	1	1	1	1	1	1	1	1	1	0	1	1	0	0	0	0	0	0	0	?	0	0	0	?	0	?	?	?	1	1	0	1	1
*S. vitreus* 50%	1	1	1	1	1	1	1	1	1	1	1	1	1	1	1	1	1	1	1	1	1	1	1	0	1	1	0	?	0	0	0	?	1	?	?	?	1	1	1	1	1
*S. vitreus* 75%	1	1	1	1	1	1	1	1	1	1	1	1	1	1	1	1	1	1	1	1	1	1	1	1	1	1	1	?	1	0	0	?	1	?	?	?	1	1	1	1	1
*S. vitreus* 100%	1	1	1	1	1	1	1	1	1	1	1	1	1	1	1	1	1	1	1	1	1	1	1	1	1	1	1	?	1	1	1	?	1	?	?	?	1	1	1	1	1
*H. fossilis* 5%	1	1	1	1	1	1	1	1	0	?	0	0	0	?	0	0	0	0	0	0	0	0	?	0	0	0	?	0	0	0	0	0	0	0	0	0	0	0	0	0	0
*H. fossilis* 10%	1	1	1	1	1	1	1	1	1	?	0	0	0	?	0	0	0	0	0	0	0	0	?	0	0	0	?	0	0	0	0	0	0	0	0	0	0	0	0	0	0
*H. fossilis* 25%	1	1	1	1	1	1	1	1	1	?	1	0	1	?	1	1	0	0	0	1	0	0	?	0	0	0	?	0	0	0	0	0	0	0	0	0	1	0	1	1	0
*H. fossilis* 50%	1	1	1	1	1	1	1	1	1	?	1	1	1	?	1	1	1	1	1	1	1	0	?	1	1	1	?	0	0	0	0	0	0	0	0	0	1	1	1	1	1
*H. fossilis* 75%	1	1	1	1	1	1	1	1	1	?	1	1	1	?	1	1	1	1	1	1	1	1	?	1	1	1	?	0	0	0	0	0	0	0	0	0	1	1	1	1	1
*H. fossilis* 100%	1	1	1	1	1	1	1	1	1	?	1	1	1	?	1	1	1	1	1	1	1	1	?	1	1	1	?	1	1	1	1	0	0	0	0	0	1	1	1	1	1
*G. morhua* 5%	1	?	1	1	1	1	0	0	0	0	0	0	0	0	?	0	?	0	0	0	0	0	0	?	0	?	?	?	?	0	0	?	0	?	?	?	0	0	?	0	?
*G. morhua* 10%	1	?	1	1	1	1	1	0	0	0	0	0	0	0	?	0	?	0	0	0	0	0	0	?	0	?	?	?	?	0	0	?	0	?	?	?	0	0	?	0	?
*G. morhua* 25%	1	?	1	1	1	1	1	1	1	1	0	0	0	0	?	0	?	0	0	0	0	0	0	?	0	?	?	?	?	0	0	?	0	?	?	?	0	0	?	0	?
*G. morhua* 50%	1	?	1	1	1	1	1	1	1	1	1	1	1	1	?	1	?	0	0	1	0	0	1	?	0	?	?	?	?	0	0	?	0	?	?	?	1	1	?	1	?
*G. morhua* 75%	1	?	1	1	1	1	1	1	1	1	1	1	1	1	?	1	?	1	1	1	1	1	1	?	1	?	?	?	?	0	0	?	0	?	?	?	1	1	?	1	?
*G. morhua* 100%	1	?	1	1	1	1	1	1	1	1	1	1	1	1	?	1	?	1	1	1	1	1	1	?	1	?	?	?	?	1	1	?	1	?	?	?	1	1	?	1	?
*O. niloticus* 5%	1	1	1	1	1	1	?	1	0	0	0	0	0	0	0	0	?	0	0	0	0	0	0	0	0	0	0	0	?	0	0	0	0	0	0	0	?	0	0	?	0
*O. niloticus* 10%	1	1	1	1	1	1	?	1	1	1	0	0	0	0	0	0	?	0	0	0	0	0	0	0	0	0	0	0	?	0	0	0	0	0	0	0	?	0	0	?	0
*O. niloticus* 25%	1	1	1	1	1	1	?	1	1	1	1	1	1	1	1	1	?	1	1	1	0	1	1	0	0	0	0	0	?	0	0	0	0	0	0	0	?	1	1	?	1
*O. niloticus* 50%	1	1	1	1	1	1	?	1	1	1	1	1	1	1	1	1	?	1	1	1	1	1	1	1	1	1	1	1	?	1	0	0	1	0	0	0	?	1	1	?	1
*O. niloticus* 75%	1	1	1	1	1	1	?	1	1	1	1	1	1	1	1	1	?	1	1	1	1	1	1	1	1	1	1	1	?	1	0	0	1	1	1	0	?	1	1	?	1
*O. niloticus* 100%	1	1	1	1	1	1	?	1	1	1	1	1	1	1	1	1	?	1	1	1	1	1	1	1	1	1	1	1	?	1	1	0	1	1	1	0	?	1	1	?	1
*B. gonionotus* 5%	1	?	1	1	1	1	1	1	0	0	0	0	0	0	0	0	0	0	0	0	0	0	?	0	0	0	0	0	0	0	0	0	0	0	0	0	?	0	0	?	0
*B. gonionotus* 10%	1	?	1	1	1	1	1	1	1	1	1	1	1	1	1	1	0	0	0	0	0	0	?	0	0	0	0	0	0	0	0	0	0	0	0	0	?	0	1	?	0
*B. gonionotus* 25%	1	?	1	1	1	1	1	1	1	1	1	1	1	1	1	1	1	1	1	1	1	1	?	1	0	1	1	1	1	0	0	0	1	0	0	1	?	1	1	?	1
*B. gonionotus* 50%	1	?	1	1	1	1	1	1	1	1	1	1	1	1	1	1	1	1	1	1	1	1	?	1	1	1	1	1	1	1	0	0	1	0	0	1	?	1	1	?	1
*B. gonionotus* 75%	1	?	1	1	1	1	1	1	1	1	1	1	1	1	1	1	1	1	1	1	1	1	?	1	1	1	1	1	1	1	0	0	1	0	0	1	?	1	1	?	1
*B. gonionotus* 100%	1	?	1	1	1	1	1	1	1	1	1	1	1	1	1	1	1	1	1	1	1	1	?	1	1	1	1	1	1	1	1	1	1	0	0	1	?	1	1	?	1
*P. parva* 5%	1	1	1	1	1	1	1	1	1	0	0	0	0	?	0	0	0	0	0	0	0	0	0	?	0	0	?	?	?	?	0	0	0	0	0	0	?	0	0	0	?
*P. parva* 10%	1	1	1	1	1	1	1	1	1	1	0	0	0	?	0	0	0	0	0	0	0	0	0	?	0	0	?	?	?	?	0	0	0	0	0	0	?	0	0	0	?
*P. parva* 25%	1	1	1	1	1	1	1	1	1	1	1	1	1	?	1	1	1	1	1	1	0	1	1	?	1	0	?	?	?	?	0	0	0	0	0	0	?	1	0	1	?
*P. parva* 50%	1	1	1	1	1	1	1	1	1	1	1	1	1	?	1	1	1	1	1	1	0	1	1	?	1	1	?	?	?	?	0	0	0	0	0	0	?	1	1	1	?
*P. parva* 75%	1	1	1	1	1	1	1	1	1	1	1	1	1	?	1	1	1	1	1	1	1	1	1	?	1	1	?	?	?	?	0	0	0	0	0	0	?	1	1	1	?
*P. parva* 100%	1	1	1	1	1	1	1	1	1	1	1	1	1	?	1	1	1	1	1	1	1	1	1	?	1	1	?	?	?	?	1	0	0	0	0	0	?	1	1	1	?
*E. lucius* 5%	1	?	1	?	?	1	0	0	0	0	0	0	0	0	0	0	0	0	0	0	0	0	0	0	0	0	?	0	?	0	0	0	0	0	0	?	?	0	?	0	0
*E. lucius* 10%	1	?	1	?	?	1	1	1	0	0	0	0	0	0	0	0	0	0	0	0	0	0	0	0	0	0	?	0	?	0	0	0	0	0	0	?	?	0	?	0	0
*E. lucius* 25%	1	?	1	?	?	1	1	1	1	1	1	1	1	1	1	1	0	0	0	0	0	0	0	0	0	0	?	0	?	0	0	0	0	0	0	?	?	1	?	1	0
*E. lucius* 50%	1	?	1	?	?	1	1	1	1	1	1	1	1	1	1	1	1	1	1	1	1	1	0	0	1	1	?	0	?	0	0	0	0	0	0	?	?	1	?	1	1
*E. lucius* 75%	1	?	1	?	?	1	1	1	1	1	1	1	1	1	1	1	1	1	1	1	1	1	1	1	1	1	?	1	?	1	0	0	0	0	0	?	?	1	?	1	1
*E. lucius* 100%	1	?	1	?	?	1	1	1	1	1	1	1	1	1	1	1	1	1	1	1	1	1	1	1	1	1	?	1	?	1	1	0	0	0	0	?	?	1	?	1	1
*P. fluviatilis* 5%	1	1	1	1	1	1	1	1	0	0	0	0	0	?	0	0	0	0	0	0	0	0	0	0	0	0	0	0	0	0	0	?	0	?	?	?	0	0	0	0	0
*P. fluviatilis* 10%	1	1	1	1	1	1	1	1	1	0	0	0	0	?	0	0	0	0	0	0	0	0	0	0	0	0	0	0	0	0	0	?	0	?	?	?	0	0	0	0	0
*P. fluviatilis* 25%	1	1	1	1	1	1	1	1	1	0	1	0	1	?	1	0	0	0	0	0	0	0	0	0	0	0	0	0	0	0	0	?	0	?	?	?	1	0	0	1	0
*P. fluviatilis* 50%	1	1	1	1	1	1	1	1	1	1	1	1	1	?	1	1	1	0	1	0	1	1	1	0	1	0	0	0	0	0	0	?	0	?	?	?	1	1	1	1	1
*P. fluviatilis* 75%	1	1	1	1	1	1	1	1	1	1	1	1	1	?	1	1	1	0	1	1	1	1	1	0	1	1	0	1	1	1	0	?	0	?	?	?	1	1	1	1	1
*P. fluviatilis* 100%	1	1	1	1	1	1	1	1	1	1	1	1	1	?	1	1	1	1	1	1	1	1	1	1	1	1	1	1	1	1	1	?	1	?	?	?	1	1	1	1	1
*B. barbus* 5%	1	1	1	1	1	1	1	0	0	0	0	0	0	0	0	0	0	0	0	0	0	0	0	0	0	0	0	0	0	0	0	0	0	0	0	0	0	0	?	?	0
*B. barbus* 10%	1	1	1	1	1	1	1	1	1	1	1	0	0	0	0	0	0	0	0	0	0	0	0	0	0	0	0	0	0	0	0	0	0	0	0	0	0	0	?	?	0
*B. barbus* 25%	1	1	1	1	1	1	1	1	1	1	1	1	1	1	1	1	1	1	1	1	0	0	0	0	0	0	0	0	0	0	0	0	0	0	0	0	1	1	?	?	0
*B. barbus* 50%	1	1	1	1	1	1	1	1	1	1	1	1	1	1	1	1	1	1	1	1	1	1	1	1	1	0	0	0	0	0	0	0	0	0	0	0	1	1	?	?	0
*B. barbus* 75%	1	1	1	1	1	1	1	1	1	1	1	1	1	1	1	1	1	1	1	1	1	1	1	1	1	1	1	1	1	0	0	0	0	0	0	0	1	1	?	?	1
*B. barbus* 100%	1	1	1	1	1	1	1	1	1	1	1	1	1	1	1	1	1	1	1	1	1	1	1	1	1	1	1	1	1	1	1	0	1	0	0	0	1	1	?	?	1
*T. tinca* 5%	1	1	1	1	1	1	1	1	1	0	0	0	0	0	0	0	0	0	0	0	0	0	0	0	0	0	0	0	0	0	0	0	0	0	0	0	0	?	0	?	?
*T. tinca* 10%	1	1	1	1	1	1	1	1	1	1	1	1	1	1	1	0	0	0	0	0	0	0	0	0	0	0	0	0	0	0	0	0	0	0	0	0	1	?	0	?	?
*T. tinca* 25%	1	1	1	1	1	1	1	1	1	1	1	1	1	1	1	1	1	0	1	1	0	0	1	0	0	0	0	0	0	0	0	0	0	0	0	0	1	?	0	?	?
*T. tinca* 50%	1	1	1	1	1	1	1	1	1	1	1	1	1	1	1	1	1	0	1	1	1	0	1	0	1	1	0	0	0	0	0	0	0	0	0	0	1	?	1	?	?
*T. tinca* 75%	1	1	1	1	1	1	1	1	1	1	1	1	1	1	1	1	1	1	1	1	1	1	1	1	1	1	0	1	0	0	0	0	0	0	0	0	1	?	1	?	?
*T. tinca* 100%	1	1	1	1	1	1	1	1	1	1	1	1	1	1	1	1	1	1	1	1	1	1	1	1	1	1	1	1	0	1	1	0	0	0	0	0	1	?	1	?	?
*H. hucho* 5%	1	1	1	1	1	1	0	0	0	0	0	0	0	0	0	0	0	0	0	0	0	0	0	0	0	0	0	0	0	0	0	0	0	0	0	0	?	0	0	?	?
*H. hucho* 10%	1	1	1	1	1	1	1	1	0	0	0	0	0	0	0	0	0	0	0	0	0	0	0	0	0	0	0	0	0	0	0	0	0	0	0	0	?	0	0	?	?
*H. hucho* 25%	1	1	1	1	1	1	1	1	1	1	1	1	1	1	0	0	0	0	0	0	0	0	0	0	0	0	0	0	0	0	0	0	0	0	0	0	?	1	0	?	?
*H. hucho* 50%	1	1	1	1	1	1	1	1	1	1	1	1	1	1	1	1	1	0	1	1	0	1	0	0	0	0	0	0	0	0	0	0	0	0	0	0	?	1	1	?	?
*H. hucho* 75%	1	1	1	1	1	1	1	1	1	1	1	1	1	1	1	1	1	1	1	1	1	1	1	1	1	1	1	1	1	1	0	0	1	1	1	0	?	1	1	?	?
*H. hucho* 100%	1	1	1	1	1	1	1	1	1	1	1	1	1	1	1	1	1	1	1	1	1	1	1	1	1	1	1	1	1	1	1	0	1	1	1	0	?	1	1	?	?
*T. thymallus* 5%	1	1	1	1	1	1	1	1	0	?	0	?	0	?	0	0	0	0	0	0	0	0	0	0	0	0	0	0	0	0	0	0	0	0	0	0	?	0	0	?	0
*T. thymallus* 10%	1	1	1	1	1	1	1	1	0	?	0	?	0	?	0	0	0	0	0	0	0	0	0	0	0	0	0	0	0	0	0	0	0	0	0	0	?	0	0	?	0
*T. thymallus* 25%	1	1	1	1	1	1	1	1	1	?	1	?	1	?	1	1	1	0	0	0	0	1	0	0	0	0	0	0	0	0	0	0	0	0	0	0	?	1	0	?	0
*T. thymallus* 50%	1	1	1	1	1	1	1	1	1	?	1	?	1	?	1	1	1	1	1	1	0	1	1	0	1	0	0	0	0	0	0	0	0	0	0	0	?	1	1	?	0
*T. thymallus* 75%	1	1	1	1	1	1	1	1	1	?	1	?	1	?	1	1	1	1	1	1	1	1	1	0	1	1	0	0	1	1	0	0	0	0	0	0	?	1	1	?	1
*T. thymallus* 100%	1	1	1	1	1	1	1	1	1	?	1	?	1	?	1	1	1	1	1	1	1	1	1	1	1	1	1	1	1	1	1	0	1	1	1	0	?	1	1	?	1
*E. caeruleum* 5%	1	1	1	1	1	1	1	?	0	0	?	?	0	0	0	0	0	0	0	?	0	0	?	0	0	0	0	0	?	0	0	0	0	0	0	0	0	0	0	0	0
*E. caeruleum* 10%	1	1	1	1	1	1	1	?	1	1	?	?	0	0	0	0	0	0	0	?	0	0	?	0	0	0	0	0	?	0	0	0	0	0	0	0	0	0	0	1	0
*E. caeruleum* 25%	1	1	1	1	1	1	1	?	1	1	?	?	1	1	1	1	1	1	1	?	0	0	?	0	0	0	0	0	?	0	0	0	0	0	0	0	1	1	1	1	1
*E. caeruleum* 50%	1	1	1	1	1	1	1	?	1	1	?	?	1	1	1	1	1	1	1	?	0	1	?	0	1	1	0	0	?	1	0	0	0	0	0	0	1	1	1	1	1
*E. caeruleum* 75%	1	1	1	1	1	1	1	?	1	1	?	?	1	1	1	1	1	1	1	?	1	1	?	1	1	1	1	1	?	1	0	0	0	0	0	0	1	1	1	1	1
*E. caeruleum* 100%	1	1	1	1	1	1	1	?	1	1	?	?	1	1	1	1	1	1	1	?	1	1	?	1	1	1	1	1	?	1	1	0	1	0	0	0	1	1	1	1	1
*O. mykiss* 5%	1	1	?	1	1	1	1	0	?	0	?	?	0	0	?	0	0	0	0	0	0	0	?	?	0	0	?	0	?	?	0	?	?	0	0	?	?	0	0	0	?
*O. mykiss* 10%	1	1	?	1	1	1	1	1	?	0	?	?	0	0	?	0	0	0	0	0	0	0	?	?	0	0	?	0	?	?	0	?	?	0	0	?	?	0	0	0	?
*O. mykiss* 25%	1	1	?	1	1	1	1	1	?	1	?	?	1	1	?	1	1	1	0	0	0	1	?	?	0	0	?	0	?	?	0	?	?	0	0	?	?	1	0	1	?
*O. mykiss* 50%	1	1	?	1	1	1	1	1	?	1	?	?	1	1	?	1	1	1	1	0	0	1	?	?	1	0	?	1	?	?	0	?	?	0	1	?	?	1	1	1	?
*O. mykiss* 75%	1	1	?	1	1	1	1	1	?	1	?	?	1	1	?	1	1	1	1	1	1	1	?	?	1	1	?	1	?	?	0	?	?	1	1	?	?	1	1	1	?
*O. mykiss* 100%	1	1	?	1	1	1	1	1	?	1	?	?	1	1	?	1	1	1	1	1	1	1	?	?	1	1	?	1	?	?	1	?	?	1	1	?	?	1	1	1	?
*P. fridmani* 5%	1	?	?	?	?	1	1	1	0	?	0	0	0	?	?	0	0	?	0	0	0	0	0	?	0	0	?	?	0	0	0	0	0	0	0	0	0	0	0	0	0
*P. fridmani* 10%	1	?	?	?	?	1	1	1	1	?	0	0	0	?	?	0	0	?	0	0	0	0	0	?	0	0	?	?	0	0	0	0	0	0	0	0	0	0	0	0	0
*P. fridmani* 25%	1	?	?	?	?	1	1	1	1	?	1	1	1	?	?	1	1	?	0	0	0	0	0	?	0	1	?	?	0	0	0	0	0	0	0	0	1	0	0	1	1
*P. fridmani* 50%	1	?	?	?	?	1	1	1	1	?	1	1	1	?	?	1	1	?	1	1	0	0	1	?	1	1	?	?	0	0	0	0	0	0	0	0	1	1	1	1	1
*P. fridmani* 75%	1	?	?	?	?	1	1	1	1	?	1	1	1	?	?	1	1	?	1	1	1	0	1	?	1	1	?	?	1	0	0	0	0	0	0	0	1	1	1	1	1
*P. fridmani* 100%	1	?	?	?	?	1	1	1	1	?	1	1	1	?	?	1	1	?	1	1	1	1	1	?	1	1	?	?	1	1	1	0	0	0	0	0	1	1	1	1	1
*N. ophidion* 5%	1	?	?	1	?	?	?	0	0	?	0	?	?	?	?	0	?	?	0	0	0	0	?	0	0	?	?	?	?	?	0	0	?	0	?	?	?	0	?	?	0
*N. ophidion* 10%	1	?	?	1	?	?	?	1	0	?	0	?	?	?	?	0	?	?	0	0	0	0	?	0	0	?	?	?	?	?	0	0	?	0	?	?	?	0	?	?	0
*N. ophidion* 25%	1	?	?	1	?	?	?	1	1	?	0	?	?	?	?	0	?	?	0	0	0	0	?	0	0	?	?	?	?	?	0	0	?	0	?	?	?	0	?	?	0
*N. ophidion* 50%	1	?	?	1	?	?	?	1	1	?	1	?	?	?	?	1	?	?	1	0	0	1	?	0	1	?	?	?	?	?	0	0	?	0	?	?	?	1	?	?	1
*N. ophidion* 75%	1	?	?	1	?	?	?	1	1	?	1	?	?	?	?	1	?	?	1	1	0	1	?	0	1	?	?	?	?	?	0	0	?	0	?	?	?	1	?	?	1
*N. ophidion* 100%	1	?	?	1	?	?	?	1	1	?	1	?	?	?	?	1	?	?	1	1	1	1	?	1	1	?	?	?	?	?	1	1	?	1	?	?	?	1	?	?	1
*S. typhle* 5%	1	?	?	?	?	?	?	0	0	?	0	?	?	?	?	0	?	?	0	?	0	0	?	0	0	?	?	?	?	?	0	0	0	0	0	?	?	0	?	?	0
*S. typhle* 10%	1	?	?	?	?	?	?	1	0	?	0	?	?	?	?	0	?	?	0	?	0	0	?	0	0	?	?	?	?	?	0	0	0	0	0	?	?	0	?	?	0
*S. typhle* 25%	1	?	?	?	?	?	?	1	1	?	1	?	?	?	?	0	?	?	0	?	0	0	?	0	0	?	?	?	?	?	0	0	0	0	0	?	?	0	?	?	0
*S. typhle* 50%	1	?	?	?	?	?	?	1	1	?	1	?	?	?	?	1	?	?	1	?	1	1	?	0	1	?	?	?	?	?	0	0	0	0	0	?	?	1	?	?	1
*S. typhle* 75%	1	?	?	?	?	?	?	1	1	?	1	?	?	?	?	1	?	?	1	?	1	1	?	1	1	?	?	?	?	?	0	0	1	1	0	?	?	1	?	?	1
*S. typhle* 100%	1	?	?	?	?	?	?	1	1	?	1	?	?	?	?	1	?	?	1	?	1	1	?	1	1	?	?	?	?	?	1	1	1	1	1	?	?	1	?	?	1
*H. erectus* 5%	1	?	?	1	?	?	?	0	0	?	0	?	?	?	?	0	?	?	0	?	0	0	?	0	0	?	?	?	?	?	0	0	0	0	0	?	?	0	?	?	0
*H. erectus* 10%	1	?	?	1	?	?	?	1	0	?	0	?	?	?	?	0	?	?	0	?	0	0	?	0	0	?	?	?	?	?	0	0	0	0	0	?	?	0	?	?	0
*H. erectus* 25%	1	?	?	1	?	?	?	1	1	?	1	?	?	?	?	0	?	?	0	?	0	0	?	0	0	?	?	?	?	?	0	0	0	0	0	?	?	1	?	?	0
*H. erectus* 50%	1	?	?	1	?	?	?	1	1	?	1	?	?	?	?	1	?	?	1	?	0	1	?	0	1	?	?	?	?	?	0	0	0	0	0	?	?	1	?	?	1
*H. erectus* 75%	1	?	?	1	?	?	?	1	1	?	1	?	?	?	?	1	?	?	1	?	1	1	?	1	1	?	?	?	?	?	0	0	0	1	1	?	?	1	?	?	1
*H. erectus* 100%	1	?	?	1	?	?	?	1	1	?	1	?	?	?	?	1	?	?	1	?	1	1	?	1	1	?	?	?	?	?	1	1	1	1	1	?	?	1	?	?	1

## Data Availability

The original contributions presented in this study are included in the article. Specifically, the coded data are available in the matrix in Table 2, and the source data from previously published developmental series can be found in the references cited in Table 1. Further inquiries can be directed to the corresponding author.

## Appendix A

Appendix A—Data submitted to the chi-square tests: observed and expected (in brackets) frequencies are distributed across three (above) or two (below) categories of three parameters: dioxygen availability, rate of anatomical development and relative hatching time.

	O_2_-poor	O_2_-rich		Medium-O_2_
Accelerated dev.	2 (0.526)	1 (1.842)		2 (2.632)
Delayed dev.	0 (0.421)	4 (1.474)		0 (2.105)
Normal dev.	0 (1.053)	2 (3.684)		8 (5.263)
	Hatching at 0–33%	Hatching at 33–66%		Hatching 66–100%
Accelerated dev.	3 (1.316)	1 (2.105)		1 (1.579)
Delayed dev.	0 (1.053)	0 (1.684)		4 (1.263)
Normal dev.	2 (2.632)	7 (4.211)		1 (3.158)
	Hatching at 0–33%	Hatching at 33–66%		Hatching 66–100%
O_2_-poor	2 (0.526)	0 (0.842)		0 (0.632)
O_2_-rich	0 (1.842)	1 (2.947)		6 (2.211)
Medium-O_2_	3 (2.632)	7 (4.211)		0 (3.158)
	O_2_-poor		O_2_-rich	
Accelerated dev.	2 (0.857)		1 (2.143)	
Delayed dev.	0 (1.143)		4 (2.857)	
	Hatching at 0–33%		Hatching 66–100%	
Accelerated dev.	3 (1.5)		1 (2.5)	
Delayed dev.	0 (1.5)		4 (2.5)	
	Hatching at 0–33%		Hatching 66–100%	
O_2_-poor	2 (0.5)		0 (1.5)	
O_2_-rich	0 (1.5)		6 (4.5)	

## References

[1] Gould S.J. (1977). Ontogeny and Phylogeny.

[2] Alberch P., Gould S.J., Oster G.F., Wake D.B. (1979). Size and shape in ontogeny and phylogeny. Paleobiology.

[3] Smith K.K. (2001). Heterochrony revisited: The evolution of developmental sequences. Biol. J. Linn. Soc..

[4] Webster M., Zelditch M.L. (2005). Evolutionary modifications of ontogeny: Heterochrony and beyond. Paleobiology.

[5] Alberch P. (1980). Heterochrony and Adaptation in the Evolution of Bolitoglossa (Amphibia, Caudata).

[6] McNamara K.J. (1989). The role of heterochrony in the evolution of spatangoid echinoids. Geobios.

[7] McNamara K.J., Minugh-Purvis N., McNamara K.J. (2002). Sequential hypermorphosis: The key to hominid evolution. Human Evolution and Developmental Change.

[8] McNamara K.J. (2012). Heterochrony: The Evolution of Development. Evol. Educ. Outreach.

[9] Broughton D.A., Collette B.B., Mc Cune A.R. (1991). Heterochrony in jaw morphology of needlefishes (Teleostei: Belonidae). Syst. Zool..

[10] Fuiman L.A. (1997). What can flatfish ontogenies tell us about pelagic and benthic lifestyles?. J. Sea Res..

[11] Fuiman L.A. (1985). Contributions of developmental characters to a phylogeny of catostomid fishes, with comments on heterochrony. Copeia.

[12] Shardo J.D. (1995). Comparative embryology of teleostean fishes. I. Development and staging of the American shad, *Alosa sapidissima* (Wilson, 1811). J. Morphol..

[13] McKinney M.L., McNamara K.J. (1991). Heterochrony: The Evolution of Ontogeny.

[14] Cangelosi A. (1999). Heterochrony and adaptation in developing neural networks. Proc. Genet. Evol. Comput. Conf..

[15] de Matos A., Suzuki R., Arita T. (2007). Heterochrony and evolvability in neural network development. Artif. Life Robot..

[16] Smith C., Wootton R.J. (2016). The remarkable reproductive diversity of teleost fishes. Fish Fish..

[17] Teletchea F., Fostier A., Kamler E., Gardeur J.N., Le Bail P.Y., Jalabert B., Fontaine P. (2009). Comparative analysis of reproductive traits in 65 freshwater fish species: Application to the domestication of new fish species. Rev. Fish Biol. Fish..

[18] Teletchea F., Fontaine P. (2010). Comparison of early-life stage strategies in 65 European freshwater fish species: Trade-offs are directed towards first-feeding of larvae in spring and early-summer. J. Fish Biol..

[19] Teletchea F. (2020). Reproductive Biology of Teleost Fishes. Robert J. Wootton and Carl Smith. Wiley Blackwell, Chichester, West Sussex, UK. 2014. Book Review. Trans. Am. Fish. Soc..

[20] Peňáz M. (1983). Ecomorphological laws and saltation in the early ontogeny of Salmonoidei. Folia Zool..

[21] Hempel G. (1979). The Early Life History of Marine Fish. The Egg Stage.

[22] Kamler E. (2002). Ontogeny of yolk-feeding fish: An ecological perspective. Rev. Fish Biol. Fish..

[23] Lecointre G., Schnell N.K., Teletchea F. (2020). Hierarchical analysis of ontogenetic time to describe heterochrony and taxonomy of developmental stages. Sci. Rep..

[24] Balon E.K. (1971). The intervals of early fish development and their terminology (A review and proposals). Věst. Česk. Spol. Zool..

[25] Balon E.K. (1975). Terminology of intervals in fish development. J. Fish. Res. Board Can..

[26] Urho L. (2002). Characters of larvae—What are they?. Folia Zool..

[27] Haug J.T. (2020). Why the term “larva” is ambiguous, or what makes a larva?. Acta. Zool..

[28] Kendall A.W., Ahlstrom E.H., Moser H.G., Moser H.G., Richards W.J., Cohen D.M., Fahay M.P., Kendall A.W., Richardson S.L. (1984). Early life history stages of fishes and their characters. Ontogeny and Systematics of Fishes Based on an International Symposium Dedicated to the Memory of E.H. Ahlstrom, 15–18 August 1983, La Jolla, California.

[29] Aral F., Şahınöz E., Doğu Z., Aral F., Doğu Z. (2011). Embryonic and Larval Development of Freshwater Fish. Recent Advances in Fish Farms.

[30] Teletchea F., Pauly D. (2024). Why do fish larvae hatch when they do?. Environ. Biol. Fishes.

[31] Hennig W. (1950). Grundzüge einer Theorie der Phylogenetischen Systematik.

[32] Hennig W. (1966). Phylogenetic Systematics.

[33] Nunn C.L., Smith K.K. (1998). Statistical analyses of developmental sequences: The craniofacial region in marsupial and placental mammals. Am. Nat..

[34] Steyer J.S. (2000). Ontogeny and phylogeny in temnospondyls: A new method of analysis. Zool. J. Linn. Soc..

[35] Bininda-Emonds O.R., Jeffery J.E., Coates M.I., Richardson M.K. (2002). From Haeckel to event-pairing: The evolution of developmental sequences. Theory Biosci..

[36] Germain D., Laurin M. (2009). Evolution of ossification sequences in salamanders and urodele origins assessed through event-pairing and new methods. Evol. Dev..

[37] Laurin M., Germain D. (2011). Developmental characters in phylogenetic inference and their absolute timing information. Syst. Biol..

[38] Roux N., Salis P., Lambert A., Logeux V., Soulat O., Romans P., Frédérich B., Lecchini D., Laudet V. (2019). Staging and normal table of postembryonic development of the clownfish (*Amphiprion ocellaris*). Dev. Dyn..

[39] Salis P., Lee S.H., Roux N., Lecchini D., Laudet V. (2021). The real Nemo movie: Description of embryonic development in *Amphiprion ocellaris* from first division to hatching. Dev. Dyn..

[40] Ghosh S.T.T., Kumar A., Balasubramanian T. (2012). Determining the level of parental care relating fanning behavior of five species of clownfishes in captivity. Indian J. Geo-Mar. Sci..

[41] Basak S., Basak B., Gupta N., Rahman S., Mohammad M., Haque M. (2014). Embryonic and Larval Development of Silver Barb (*Barbodes gonionotus*) in a Mobile Hatchery under Laboratory Condition. IOSR J. Agric. Vet. Sci. (IOSR-JAVS).

[42] Rainboth W.J. (1996). Fishes of the Cambodian Mekong. FAO Species Identification Field Guide for Fishery Purposes.

[43] Nasution N.A., Machrizal R. (2021). Bioecological aspect of lamasi (*Barbonymus gonionotus*) in mailil rever labuhanbatu district, Indonesia. JPBIO (J. Pendidik. Biol.).

[44] Krupka I. (1988). Early development of the barbel (*Barbus barbus* (Linnaeus, 1758)). Práce Ust. Ryb. A Hydrobiol..

[45] Kottelat M., Freyhof J. (2007). Handbook of European Freshwater Fishes.

[46] Pospisilova A., Brejcha J., Miller V., Holcman R., Sanda R., Stundl J. (2019). Embryonic and larval development of the northern pike: An emerging fish model system for evo-devo research. J. Morphol..

[47] Paine M.D., Balon E.K. (1984). Early development of the rainbow darter, *Etheostoma caeruleum*, according to the theory of saltatory ontogeny. Environ. Biol. Fishes.

[48] Harding J.M., Burky A.J., Way C.M. (1998). Habitat preferences of the Rainbow Darter, *Etheostoma caeruleum*, with regard to microhabitat velocity shelters. Copeia.

[49] Hlohowskyj I., Wissing T.E. (1987). Seasonal changes in low oxygen tolerance of fantail, *Etheostoma flabellare*, rainbow, *E. caeruleum*, and greenside, *E. blennioides*, darters. Environ. Biol. Fishes.

[50] Hall T.E., Smith P., Johnston I.A. (2004). Stages of embryonic development in the Atlantic cod (*Gadus morhua*). J. Morphol..

[51] Thorarensen H., Gústavsson A., Gunnarsson S., Árnason J., Steinarsson A., Björnsdóttir R., Imsland A.K.D. (2017). The effect of oxygen saturation on the growth and feed conversion of juvenile Atlantic cod (*Gadus morhua* L.). Aquaculture.

[52] Puvaneswari S., Marimuthu K., Karuppasamy R., Haniffa M.A. (2009). Early Embryonic and Larval Development of Indian Catfish, *Heteropneustes fossilis*. EurAsian J. Biosci..

[53] Froese R., Pauly D. (2000). FishBase 2000: Concepts, design and data sources. ICLARM, Los Baños, Laguna, Philippines. 344p. https://www.fishbase.se/.

[54] Schneider R.F., Woltering J.M., Adriaens D., Roth O. (2023). A comparative analysis of the ontogeny of syngnathids (pipefishes and seahorses) reveals how heterochrony contributed to their diversification. Dev. Dyn..

[55] Peňáz M., Prihoda J. (1981). Reproduction and early development of *Hucho hucho*. Acta Sc. Nat. Brno..

[56] Braga Goncalves I., Ahnesjö I., Kvarnemo C. (2016). Evolutionary ecology of pipefish brooding structures: Embryo survival and growth do not improve with a pouch. Ecol. Evol..

[57] Knight A.E. (1963). The Embryonic and Larval Development of the Rainbow Trout. Trans. Am. Fish. Soc..

[58] Fujimura K., Okada N. (2007). Development of the embryo, larva and early juvenile of Nile tilapia *Oreochromis niloticus* (Pisces: Cichlidae). Developmental staging system. Dev. Growth Differ..

[59] Alix M., Chardard D., Ledoré Y., Fontaine P., Schaerlinger B. (2015). An alternative developmental table to describe non-model fish species embryogenesis: Application to the description of the Eurasian perch (*Perca fluviatilis* L. 1758) development. EvoDevo.

[60] Chen J.Y., Zeng C., Cobcroft J.M. (2023). Year-Round Spawning, Filial Cannibalism, and Embryonic and Larval Development of the Coral Reef Fish Orchid Dottyback, *Pseudochromis fridmani*. Fishes.

[61] Mies M., Güth A.Z., Scozzafave M.S., Sumida P.Y.G. (2014). Spawning behaviour and activity in seven species of ornamental dottybacks. J. Zoo Aquar. Res..

[62] Zhu D., Yang K., Sun N., Wang W., Zhou X. (2018). Embryonic and larval development of the topmouth gudgeon, *Pseudorasbora parva* (Teleostei: Cyprinidae). Zoologia.

[63] McElman J.F., Balon E.K. (1979). Early ontogeny of walleye, *Stizostedion vitreum*, with steps of saltatory development. Environ. Biol. Fishes.

[64] Peňáz M. (1975). Early development of the grayling *Thymallus thymallus* (Linnaeus, 1758). Acta Sci. Nat. Acad. Sci. Bohemoslov.—Brno.

[65] Keith P., Poulet N., Denys G., Changeux T., Feunteun E., Persat H. (2020). Les poissons d’eau douce de France.

[66] Peňáz M., Wohlgemut E., Hamáčková J., Kouřil J. (1981). Early ontogeny of the tench, *Tinca tinca* (Linnaeus, 1758). I. Embryonic period. Folia Zool..

[67] Peňáz M., Wohlgemut E., Hamáčková J., Kouřil J. (1982). Early ontogeny of the tench, *Tinca tinca* (Linnaeus, 1758). II. Larval period. Folia Zool..

[68] Yilmaz F. (2002). Reproductive biology of the tench *Tinca tinca* (L., 1758) inhabiting Porsuk Dam Lake (Kutahya, Turkey). Fish. Res..

[69] Kunz Y.W. (2004). Developmental Biology of Teleost Fishes.

[70] von Baer K.E. Über Entwickelungsgeschichte der Thiere. Beobachtung und reflexion. [On the Developmental History of the Animals. Observations and Reflections] (Bei den Gebrüdern Bornträger, Königsberg, 1828. http://www.biodiversitylibrary.org/item/28306.

[71] Nelson G., Platnick N.I. (1991). Three-taxon statements: A more precise use of parsimony?. Cladistics.

[72] Cao N., Zaragüeta i Bagils R., Vignes-Lebbe R. (2007). Hierarchical representation of hypotheses of homology. Geodiversitas.

[73] Rineau V., Zaharias P. (2025). Agatta (Version 0.9.1) [Software]. Github. https://github.com/vrineau/Agatta.

[74] Nelson G., Ladiges P.Y. (1992). Information Content and Fractional Weight of Three-Item Statements. Syst. Biol..

[75] Rineau V., Zaragüeta R., Bardin J. (2021). Information Content of Trees: Three-taxon Statements Inference Rules and Dependency. bioRxiv.

[76] Mahbub M., Wahab Z., Reaz R., Saifur Rahman M., Bayzid S. (2021). wQFM: Highly accurate genome-scale species tree estimation from weighted quartets. Bioinformatics.

[77] Archie J.W. (1989). Homoplasy Excess Ratios: New Indices for Measuring Levels of Homoplasy in Phylogenetic Systematics and a Critique of the Consistency Index. Syst. Biol..

[78] Farris J.S. (1989). The Retention Index and the Rescaled Consistency Index. Cladistics.

[79] Kitching I.J., Forey P.L., Humphries C.J., Williams D.M. (1998). Cladistics: The Theory and Practice of Parsimony Analysis.

[80] Rineau V., García-Medrano P., Zeitoun V., Zaragüeta-i-Bagils R., Ashton N., Arzarello M., Ollé A., Lamotte A., Peretto C., Moncel M.H. (2026). Technological innovations and regional diversity in Western Europe at ca. the MIS 11 threshold: A cladistic approach. Quat. Sci. Adv..

[81] Minelli A., Pradeu T. (2014). Toward a Theory of Development.

[82] Lecointre G., du Crest A., Valkovic M., Ariew A., Desmond H., Huneman P., Reydon T.A.C. (2023). Darwinian/Hennigian Systematics and Evo-Devo: The missed Rendez-Vous. Evolutionary Thinking Across Disciplines. Problems and Perspectives in Generalized Darwinism.

[83] Lecointre G. (2026). Development is an evolutionary phenomenon. C.R. Biol..

[84] Teletchea F., Lecointre G. (2026). What is a mature larva?. Cybium.

[85] Keckeis H., Baeur-Nemeschkal E., Kamler E. (1996). Effects of reduced oxygen level on the mortality and hatching rate of *Chondrostoma nasus* embryos. J. Fish Biol..

[86] Czerkies P., Kordalski K., Golas T., Krysinski D., Luczynski M. (2002). Oxygen requirements of whitefish and vendace (Coregonidae) embryos at final stages of their development. Aquaculture.

[87] Fuiman L.A., Fuiman L.A., Werner R.G. (2002). Special considerations of eggs and larvae. Fishery Science—The Unique Contributions of Early Life Stages.

[88] Kim K.Y., Ko M.H., Liu H., Tang Q., Chen X., Miyazaki J., Bang I.C. (2013). Phylogenetic relationships of *Pseudorasbora*, *Pseudopungtungia*, and *Pungtungia* (Teleostei; Cypriniformes; Gobioninae) inferred from multiple nuclear gene sequences. Biomed. Res. Int..

[89] Gray R.E. (2026). Reconstructing the Past: Scientific Reasoning in the Historical Sciences Through the Lens of Endosymbiotic Theory. Sci. Educ..

[90] Santini S., Polacco G. (2006). Finding Nemo: Molecular phylogeny and evolution of the unusual life style of anemonefish. Gene.

[91] Litsios G., Salamin N. (2014). Hybridisation and diversification in the adaptive radiation of clownfishes. BMC Evol. Biol..

[92] Li J., Chen X., Kang B., Liu M. (2015). Mitochondrial DNA Genomes Organization and Phylogenetic Relationships Analysis of Eight Anemonefishes (Pomacentridae: Amphiprioninae). PLoS ONE.

[93] Nguyen H.T.T., Dang B.T., Glenner H., Geffen A.J. (2020). Cophylogenetic analysis of the relationship between anemonefish *Amphiprion* (Perciformes: Pomacentridae) and their symbiotic host anemones (Anthozoa: Actiniaria). Mar. Biol. Res..

[94] Dhaneesh K.V., Ajith Kumar T.T., Shunmugaraj T. (2009). Embryonic Development of Percula Clownfish, *Amphiprion percula* (Lacepede, 1802). Middle-East J. Sci. Res..

[95] Breder C.M., Rosen D.E. (1966). Modes of Reproduction in Fishes.

